# Endocannabinoid signaling in social functioning: an RDoC perspective

**DOI:** 10.1038/tp.2016.169

**Published:** 2016-09-27

**Authors:** D S Karhson, A Y Hardan, K J Parker

**Affiliations:** 1 Center for Interdisciplinary Brain Sciences Research, Department of Psychiatry and Behavioral Sciences, Stanford University School of Medicine, Stanford, CA, USA; 2 Division of Child and Adolescent Psychiatry, Department of Psychiatry and Behavioral Sciences, Stanford University School of Medicine, Stanford, CA, USA

## Abstract

Core deficits in social functioning are associated with various neuropsychiatric and neurodevelopmental disorders, yet biomarker identification and the development of effective pharmacological interventions has been limited. Recent data suggest the intriguing possibility that endogenous cannabinoids, a class of lipid neuromodulators generally implicated in the regulation of neurotransmitter release, may contribute to species-typical social functioning. Systematic study of the endogenous cannabinoid signaling could, therefore, yield novel approaches to understand the neurobiological underpinnings of atypical social functioning. This article provides a critical review of the major components of the endogenous cannabinoid system (for example, primary receptors and effectors—Δ9-tetrahydrocannabinol, cannabidiol, anandamide and 2-arachidonoylglycerol) and the contributions of cannabinoid signaling to social functioning. Data are evaluated in the context of Research Domain Criteria constructs (for example, anxiety, chronic stress, reward learning, motivation, declarative and working memory, affiliation and attachment, and social communication) to enable interrogation of endogenous cannabinoid signaling in social functioning across diagnostic categories. The empirical evidence reviewed strongly supports the role for dysregulated cannabinoid signaling in the pathophysiology of social functioning deficits observed in brain disorders, such as autism spectrum disorder, schizophrenia, major depressive disorder, posttraumatic stress disorder and bipolar disorder. Moreover, these findings indicate that the endogenous cannabinoid system holds exceptional promise as a biological marker of, and potential treatment target for, neuropsychiatric and neurodevelopmental disorders characterized by impairments in social functioning.

## Introduction

Social functioning impairments are frequently cited as core features of neuropsychiatric and neurodevelopmental disorders, yet progress in biomarker identification and development of targeted pharmacotherapies has been extremely limited. Delays in progress are attributed to social deficit heterogeneity and use of the Diagnostic and Statistical Manual of Mental Disorders (DSM) for investigational research.^1, 2, 3^ The DSM is a categorical classification system intended to provide clinicians with a common language for diagnosis, but its use in empirical research confers a ‘top-down' design. Clinicians categorize patients phenomenologically using the DSM, then researchers attempt to identify pathophysiological mechanisms in patient-participants. However, congruence in phenomenology between patients does not necessarily signify similarity in endophenotypes or genotypes, confounding explicit links to pathology.^3, 4^ To address the disparity in our current understanding of brain dysfunction with clinical phenomenology, a paradigm shift towards a dimensional ‘bottom-up' design in research has been adopted. The Research Domain Criteria (RDoC) is a translational research approach that utilizes a matrix framework to facilitate studies that cut across diagnostic boundaries by probing functional constructs associated with neural circuits. The RDoC provides a comprehensive infrastructure to interrogate social functioning impairments and strongly emphasizes the appropriate experimental classification of patients to support direct translation of research findings for clinical use.^2^ Social competence is an emergent property, inter- and intra-dependent on functional integrity of three of the RDoC domains: negative valence systems, positive valence systems and cognitive systems. Initial stages of social interaction require overcoming negative valence systems (for example, fear, anxiety) to initiate the interaction and are reinforced by positive valence systems (for example, reward learning, reward valuation). Cognitive Systems (that is, attention, perception, working memory) guide the exchange after social interaction has commenced. Social process systems (that is, affiliation and attachment, social communication, perception of self and others) exert supramodal control to coordinate germane practices. Dysfunction in one construct intrinsically affects social information processing and impacts the ability to function typically. In addition, the RDoC framework encourages experimental inquiry at multiple levels of analysis (that is, genes, molecules, cells, circuits, physiology, behavior) to foster research within and across constructs, which fundamentally highlights neuromodulatory systems that operate in multiple constructs, such as the cannabinoid system. The functional heterogeneity of the endogenous cannabinoid system recapitulates the diversity of social functioning abilities in neuropsychiatric and neurodevelopmental disorder patients and parallels dimensional analysis of the RDoC.

The first reports on cannabinoid involvement in social functioning are from the nineteenth century psychiatrist Dr. Jacques Moreau de Tours for the treatment of distributed ‘neurological dysregulation' and ‘social alienation'.^5, 6, 7^ Dr. Moreau noted similarities from experiences in healthy humans after ingesting North African hashish (which contains ‘very high concentration of THC [Δ9-tetrahydrocannabinol]'^5^) with the diversity of behaviors in neurological dysfunction.^6^ Moreover, first-person reports from hashish users detail traits that neatly map onto RDoC constructs: for example, ‘fluctuations of emotions' (negative valence), extreme ‘happiness, excitement' (positive valence), ‘errors of time and space…and illusions and hallucinations'^6^ (arousal/regulatory), ‘irresistible impulses…and dissociation of ideas' (cognitive domain).^8, 9^ However, current research is focused on the use of inhibitors of endogenous cannabinoid degradation (to enhance signaling) in the study of reward-related processing in social interactions.^10, 11, 12, 13, 14^ The present article reviews the basic biology of the endogenous cannabinoid system and the roles of its relevant components across each RDoC construct as it pertains to social functioning. Data reviewed within each RDoC construct are applicable to multiple neuropsychiatric and neurodevelopmental disorders, the most notable of which are highlighted as exemplars at the conclusion of each section. With development of diagnostic assessments and treatment options for social functioning deficits limited due to inherent phenotypic heterogeneity, methodical research on the endogenous cannabinoid system has high potential to provide inroads in identifying underlying mechanisms shared across disorders.

## Endocannabinoid signaling: effectors in neuropsychiatric and neurodevelopmental disorders

### Receptors

Cannabinergic effects is chiefly mediated by stimulation of cannabinoid receptors, type 1 and 2 (CB1R and CB2R),^15^ which are distributed throughout the central nervous system^8, 16^ (see Table 1). Both types are seven-transmembrane G-protein-coupled receptors. These receptors stimulate G_i/o_ proteins in the regulation of ion channels, inhibit adenylyl cyclase, which through downstream signaling increase cyclic AMP in the modulation of neurotransmission, activate protein kinase A to govern cellular function and regulate mitogen-activated protein kinases in control of transcriptional factors.^8, 17, 18^ Mainly expressed in presynaptic axonal segments of inhibitory and excitatory neurons of the brain, CB1Rs are implicated in regulation of synaptic strength.^16, 17^ CB1Rs control the probability of neurotransmitter release at glutamate and γ-aminobutyric acid (GABA) synapses, mediating GABA suppression or glutamate release and reuptake,^18, 19^ thereby contributing to the homeostatic maintenance of the brain's excitatory-inhibitory balance.^17, 20, 21^ CB1R activation also suppresses release of serotonin, dopamine, acetylcholine and noradrenaline,^18, 21^ thereby mediating the characteristic cognitive and antidepressant effects.^16, 18^ CB2Rs, in contrast, were initially considered a peripheral nervous system receptor as they were originally from immune cells and gene expression levels were highest in inflammatory responses.^15, 22^ The presence of brain CB2Rs was first detected after harm, insult or neuroinflammation, which breaks down the blood–brain barrier and allows for non-neuronal immunocytes to infiltrate the central nervous system, thereby increasing CB2R expression.^23^ Accordingly, expression levels of brain CB2R represent brain health,^22, 23^ as very low expression indicates a fit brain.^23, 24^ At homeostasis, low CB2R expression^25^ is observed in cell bodies and dendrites of neural progenitor cells, neurons, oligodendrocytes, astrocytes^23, 26^ and stimulated microglia—‘resident macrophages of the central nervous system'.^22^ Activation of CB2Rs upregulates cell-surface factors in regeneration and degeneration, functions in migration and proliferation, as well as neural cell maturation and survival,^16, 23, 27^ but the underlying mechanisms remain unclear.

### Ligands

Elucidation of the delta-9 double bond in plant-derived cannabinoid (phytocannabinoid), Δ9-tetrahydrocannabinol (THC), in the early 1960s instigated the search for endogenously synthesized cannabinoids (endocannabinoids) and led to the detection of anandamide (AEA) and 2-arachidonoylglycerol (2-AG).^65, 66^ Each cannabinoid class and type is distinct in mechanisms of action, behavioral profiles and action in neural circuits. Phytocannabinoids are produced in secretory cells of glandular trichomes at high concentrations in unfertilized female Cannabis flowers. THC is a low potency partial agonist at CB1R and CB2R.^8, 65^ The non-psychoactive analog of THC, cannabidiol (CBD), is of burgeoning clinical interest for its very low efficacy and partial agonism at CB1R and CB2R. At low molar concentrations, CBD acts as an antagonist or inverse agonist (particularly at CB2R) to limit THC effects and contributes to the upregulation of endogenous cannabinoid signaling.^67^ Clinically, CBD is a neuroprotectant used as an anti-inflammatory, anticonvulsant, antiepileptic and antipsychotic.^8^ CBD's effects are attributed to the full agonism it exhibits for non-cannabinoid G-protein-coupled receptors (for example, peroxisome proliferator-activated and nuclear receptors) and interactions with serotonergic, adenosinergic and vanilloid systems.^8, 15^ Notably, the most efficacious therapeutics are observed with formulations combining THC and CBD (two to four times greater than single cannabinoid preparations^68^) as CBD enhances the therapeutic action of THC by potentiating its psychotropic effects, augmenting THC tolerability, and widening the therapeutic window.^67^ Enhanced efficacy is attributed to the entourage effect,^65, 68^ the synergistic action of the more than 480 biologically active and inactive compounds in Cannabis. It is likely that the as-yet identified major and minor cannabinoids in plant extracts may further enhance the therapeutic benefit observed with THC and CBD through improved stimulation of the endogenous cannabinoid system.^65^

Endocannabinoids (eCBs) are small (<400 Da) lipophilic activity-dependent retrograde messengers in the brain. Produced post-synaptically ‘on demand' through *de novo* synthesis from membrane phospholipids in response to increased intracellular Ca^2+^ via depolarization,^69^ eCBs act presynaptically to inhibit GABA and glutamate release.^17^ The physiological endpoints of AEA and 2-AG are comparable to THC,^69^ but are functionally and temporally distinct from one another. AEA has high binding affinity (CB1R: *K*_i_=239 nmol l^−1^, CB2R: 440 nmol l^−1^),^70^ but low efficacy for cannabinoid receptors. It is most active during steady-state conditions and regulates basal synaptic neurotransmission. Data from rodent autism spectrum disorder (ASD) models demonstrate that genetic mutations in the neuroligin gene disrupt tonic eCB signaling^71^ and intimate the importance of eCB signaling mode (tonic or phasic) in neurodevelopment and neuropsychiatric disorders. 2-AG is the more prevalent eCB (~4 nmol g^−1^ in brain tissue vs AEA at <100 pmol g^−1^)^70^ and regulates synaptic plasticity.^17, 69^ It is representative of ‘phasic' signaling evoked in sustained depolarization. The greater overall efficacy and binding affinity of 2-AG (CB1R: *K*_i_=3424nmol l^−1^, CB2R: 1194 nmol l^−1^),^70^ particularly at CB2R,^72^ is likely related to the enhanced native tone and role in adaptive response. Inactivation of AEA and 2-AG is primarily through enzymatic degradation. AEA is transported across the transmembrane proteins^47, 73^ and hydrolyzed via fatty acid amide hydrolase (FAAH),^47^ whereas 2-AG can be hydrolyzed by FAAH or monoacylglycerol lipase.^74^

## Endocannabinoid signaling in social functioning: an RDoC perspective

The endocannabinoid system provides a consummate model to examine social functioning deficits across multiple clinical populations. The distribution of eCB components is congruent with affected neuroanatomy in neuropsychiatric and neurodevelopmental disorders with social functioning impairments (see Table 1). Changes in eCB components and function contribute to impairments in RDoC constructs critical to social behavior, resulting in a spectrum of atypical social endophenotypes. The next sections review the role of the eCB system in RDoC constructs subserving social functioning and which are characteristically observed as affected in neuropsychiatric and neurodevelopmental disorders.

## eCB signaling and negative valence systems

### Anxiety

Anxiety, fear, and stress as terms are often semantically interchangeable, though each is independently characterized by the nature of the eliciting event and magnitude of response.^28, 29^ Anxiety is elicited by putatively dangerous, uncertain, imminent situations or events that lead to an acute behavioral response in preparation for threats to individual integrity that is disproportional in intensity or chronicity.^29^ Anxiety is physiologically manifested as an exaggerated startle response, increased muscle tension, decreased motion, avoidance behaviors and autonomic hyperactivity. With regard to social functioning, pathological anxiety can impede competent functioning in the absence of triggering stimuli. Major components of the neurocircuitry mediating anxiety, such as the prefrontal cortex (PFC), hippocampus, amygdala and hypothalamus are rich in CB1R and CB2R expression (see Table 1). In animal models, genetic deletion of CB1R increases anxiety-like behaviors, but only under highly aversive conditions, whereas deletion of CB2R modulate vulnerability to anxiogensis.^30, 75^ Overexpression of CB2Rs in mouse models increases resistance to anxiogenic stimuli, mediated by increased 2-AG and GABA signaling.^30^ Exogenous cannabinoids exert an inverted U-shaped effect on anxiety, that is both anxiolytic and anxiogenic, dependent on concentration and context.^76^ Acute administration of THC and CBD in preclinical models and patient populations commonly produces an anxiolytic response, likely due to inhibition of the glutamatergic firing in neural networks involved in anxiety.^28, 29^ Similarly, eCB signaling, such as blockade of AEA hydrolysis^77, 78^ or inhibition of monoacylglycerol lipase^8^ also elicits an anxiolytic response. Anxiogensis can be observed via eCB signaling suppression of glutamate outflow in the hippocampus and periaqueductal gray, as well as in its inhibition of corticolimbic release of noradrenaline, dopamine and serotonin.^30, 76^ These data are pertinent to a myriad of neurodevelopmental and neuropsychiatric disorders (for example, posttraumatic stress disorder, depression, schizophrenia, obsessive compulsive disorder, ASD, bipolar disorder, phobias and so on), but further study is needed to clarify how best to leverage neurocircuitry modulation with precision to ensure anxiolytic function of cannabinergic pharmacologic interventions and the potential restoration of homeostatic eCB signaling.

### Chronic stress

Stress is an adaptive response to a specific internal or external stressor in preparation for injury or threat. Definitions of stress emphasize the physiological and emotional consequences to relate acute parallel activation of autonomic and endocrine systems. Chronic stress is a response to persistent stressors, like social threat or defeat, which reduces social motivation and social interactions by intensifying fear towards (an emotional reaction) and avoidance (anxiogenic behavior) of unknown conspecifics.^32, 79^ Across development, protracted exposure to stressors imposes a progressive pattern of dysfunction in social functioning, beginning as asociality and culminating in antisociality.^79^ Persistent activation (>4 weeks, ≤48 weeks) of stress neurocircuitry breaks down homeostatic balance^32, 33^ and creates a 'hypocannabinergic state' through downregulation of CB1R expression in the hippocampus, hypothalamus, striatum and dorsal root ganglion.^33, 34^ No significant change in CB2R expression was observed in three separate mouse lines, following exposure to chronic stressors, but without analogous data in humans, few conclusions can be made about CB2R function under chronic stress.^25^ Alterations in CB1R expression are concurrent with changes in eCB signaling in the hippocampus, striatum, dorsal raphe nucleus, hypothalamus, nucleus accumbens (NAc), PFC (at GABAergic terminals) and the amygdala. In the absence of stressors, AEA tone suppresses activity of the hypothalamic–pituitary–adrenal (HPA) axis (the chief modulator of the stress response^80, 81^) and disruption of this suppression initiates HPA-axis activation.^35, 81^ Chronic stressor exposure impairs AEA signaling and elevates 2-AG content within the amygdala (and increases glucocorticoid hormone secretion), hippocampus, hypothalamus and PFC,^33^ likely due to altered FAAH and monoacylglycerol lipase-mediated hydrolysis, as the synthesis of these eCBs is unimpaired.^34, 82^ Glucocorticoid feedback inhibition of HPA-axis activity is suggested to occur on two temporal scales, rapidly via the PFC and 2-AG tone enhancement, or less rapidly via negative feedback inhibition at the paraventricular nucleus of the hypothalamus.^34, 82^ Glucocorticoids then normalize the amygdalar AEA levels to support basal HPA function.^34^ The enhancement of 2-AG signaling is perceived as an attempt to habituate to the chronic stress exposure and may be crucial in understanding how to properly engage eCB signaling in the modulation of the HPA axis to guarantee a positive adaptive response.^34^ However, the presence of a mechanism to examine maladaptive responses to chronic stress exposure highlights novel areas for social deficits in which exposure to a chronic stressor is unavoidable (for example, social anxiety and mood disorders).

## eCB signaling and positive valence systems

### Reward learning

Reward attainment is one of the only RDoC constructs to explicitly detail eCBs as candidate modulators of reward learning, valuation and processing.^43^ Recent data linking reward and social neuropeptides with eCBs highlight a novel, potential intervention specific to social functioning deficits.^13^ Adaptive reinforcement of social interactions requires long-term synaptic plasticity at excitatory synapses of the NAc and is dependent on oxytocin (OT), a neuropeptide that regulates prosocial behavior and its dysregulation has been implicated in social impairments.^83, 84^ In the examination of neural circuits of socialization, Wei *et al.*^13^ demonstrated an obligatory role of AEA in a socially isolated rodent model. Salience and reward were modulated by AEA mobilization in the NAc and hippocampus via activation of OT receptors by endogenously released OT following social contact. Additional experiments to control for the anxiogenic induction of AEA release during stressful social situations found the observed effects were independent of the stress response and specific to social reward.^13^ Data suggest that OT acts as a social reinforcement signal to induce long-term depression in medium spiny neurons and requires specific activation of CB1Rs by AEA to regulate social incentive salience. These preclinical data provide insight into the mechanisms associated with OT in social functioning in clinical populations. If improvement in social functioning requires OT-evoked AEA signaling as data suggest, then enhancement of OT to improve social deficits must be sufficient to engage AEA mobilization for proper functioning of the neural circuit. Researchers have advocated that these findings are of particular relevance to ASD, although these results can also be broadly applied to neuropsychiatric disorders characterized by dysregulated OT and impaired social functioning (for example, schizophrenia and mood, anxiety, or personality disorders^85, 86^).

### Social motivation

Social motivation leads to enhancement of social development by promoting social interactions. Abnormal motivation precludes proficient social interaction and has negative consequences on the acquisition of higher-order social abilities.^37^ The inability to separate motivation from social interest has contributed to the assumption that deficits in social functioning are a cause of disrupted social interest, as opposed to a consequence of them.^37^ Motivation is necessary to attend to social information, seek and engage in social interaction, as well as foster and maintain social bonds, whereas interest does not necessitate action. Individual differences in social motivation are associated with alterations in the activity of neurocircuitry that overlap with reward learning; high social motivation is correlated with enhanced activation of the amygdala and orbital frontal cortex, whereas weaker activation is related to lower social motivation. Opioid and dopaminergic neurons in the ventral tegmental area have complementary roles in motivated behaviors and require eCB signaling to fine-tune dopamine release in incentive-related reward learning.^40, 52^ Concomitantly, enhanced motivation is observed following CB1R stimulation and with increased AEA signaling in the amygdala, NAc and dorsal striatum and 2-AG in the NAc.^38, 41, 42^ Diminished motivation is associated with CB1R blockade.^38, 39^ Wei *et al.*^87^ suggest increased AEA content in the forebrain in animal models of ASD can ameliorate social motivation deficits. Extending previous results in rats in which modulation of CB1R or genetic removal of FAAH negatively impacted social interactions,^79, 88^ researchers examined the influence of AEA on social behaviors via FAAH modulation.^13^ A social approach task and elevated plus maze were used to assess the effects of increased AEA (via FAAH inhibition) on social motivation in BTBR mice (an idiopathic ASD model with known deficits in social approach, reciprocal social interaction, and juvenile play) and Fragile X Syndrome mice (a syndromic ASD model with persistent social approach deficits). Increased AEA signaling in the forebrains of ASD mouse models correlated with increased time in the social chamber and preferential interaction with novel animals compared with untreated and control mice.^87^ Effects were not associated with decreased anxiogenic responses, as no change in behavior on the elevated plus maze was observed. Improved prosocial behaviors were ‘generalizable'^87^ and suggest common neural circuitry of social motivation between idiopathic and syndromic ASD.

## eCB signaling and cognitive systems

### Declarative memory

Declarative memory (that is, encoding, consolidation, storage and retrieval of factual information^44^) supports social interactions by providing biographical and episodic recall.^49^ It functions as a store of experience-outcomes and integrates previous conclusions with new input, as well as emotion, motivation and perception.^44^ Stimulation of cannabinoid receptors in hippocampal circuits diminishes glutamate release to below-threshold levels, inhibiting long-term potentiation necessary for encoding.^89, 90^ In animal models, stimulation of receptors before or after learning induces impaired performance on water maze, contextual fear conditioning and object recognition memory assessments.^89, 91^ In humans, deficits were not observed in the retrieval of previously stored information, and learning impairments were transient as after a 3-month abstinence from phytocannabinoids, deficits were no longer observed.^8, 92^ Importantly, cannabinergic modulation of cognitive effects can vary on the basis of the use of exogenous or endogenous cannabinoids. AEA and 2-AG are both robust modulators of early-stage acquisition, consolidation and extinction,^49, 66, 90^ but it is the enhancement of 2-AG that is correlated with disrupted encoding in spatial memory.^51, 93, 94^ An abundance of evidence demonstrates transient, dose-dependent THC-induced memory impairments (with a tolerance effect in heavy users) and the contrasting absence of memory deficits following CBD administration. Data also suggest that CBD is protective against THC-induced impairments in episodic and spatial memory.^65, 66, 92^ Explicit control of eCB signaling in declarative memory for social functioning deficits must account for individual differences in abilities. Brain disorders characterized by difficulties in uncontrolled recall (for example, ASD, posttraumatic stress disorder, phobia disorders) may benefit from control over extinction, whereas disrupting short-term memory consolidation may be advantageous for other disorders (for example, OCD, anxiety disorders).

### Working memory

More immediate information processing involves working memory, which actively maintains and updates relevant information, but is capacity-limited.^50^ In social interactions, working memory tracks social information, like the characteristics of, or relationships among, people necessary to competently socially interact. THC exposure in humans negatively impacts working memory via CB1R activation and inhibition of AEA reuptake.^48, 51^ Correspondingly, rodent models with upregulated CB1R expression in the PFC, as well as CB1R knockout mice, demonstrate changes in cognitive flexibility. Low doses of CB1R antagonists improved task switching (a measure of cognitive flexibility) and inhibitory control via inhibition of PFC glutamatergic activity, whereas CB1R agonists increased impulsive behaviors. A neuroimaging study^90^ suggests that THC impacts activity in cerebral inhibition response circuits causing increased hyperactivity in the PFC and anterior cingulate cortices. Acute administration of THC reduces response inhibition^49, 89^ (that is, increases behavioral impulsivity) and causes hyperactivity at dopaminergic synapses in the PFC.^90^ Data from animal models suggest that cannabinoid signaling interacts with dopaminergic, GABAergic and glutamatergic pathways to mediate behavioral changes. Data in FAAH knockout mice demonstrate that AEA-biased tone improves acquisition in working memory tasks, but the effects are transient and do not persist into later trials.^65, 66^ This evidence suggests that strategies to improve components of working memory (for disorders such as prosopagnosia, schizophrenia and/or depression) should focus on control of AEA tone and local modulation of 2-AG signaling.

## eCB signaling and social processing systems

### Affiliation and attachment

Maternal care is a newborn's first social experience and variation in maternal care has been shown to dramatically influence social development.^56, 57^ Mouse pups that receive intensive maternal care demonstrate enhanced maternal behavior in adulthood demonstrating the enduring effects of maternal care on adult social functioning.^95, 96^ Disruption of the mother–infant bond has lasting consequences on neuroendocrine and cognitive functioning, increasing the risk for subsequent psychopathology.^56^ OT is a primary regulator of social behavior,^53, 83, 84^ particularly in maternal care,^53, 54^ and is associated with eCB signaling.^57, 95, 97^ Schechter *et al.*^57, 95^ performed several studies examining eCB and OT signaling in maternal care and demonstrated that genetic ablation of CB1R negatively affected maternal care. This impairment correlated with decreased hippocampal OT receptor expression and increased hippocampal levels of 2-AG.^54, 57, 95^ Results are consistent with observations from socially isolated animals. These studies demonstrated that region-specific CB1R expression in the supraoptic nucleus of socially isolated animals can reduce social play and social interaction compared with those that were pair-housed and handled daily.^36, 55^ Changes in receptor density affect GABAergic and glutamatergic input to OT-synthesizing neurons for mobilization of OT release,^55, 57^ which is necessary for social bonding. Valproate-exposed rats, another animal model of social deficits used in ASD research, demonstrate similarly reduced sociability, but changes in CB1R expression were also correlated with reduced hippocampal 2-AG expression.^98^ Moreover, after social play, increased AEA and 2-AG levels in the NAc and the amygdala were observed.^12, 88^ These data demonstrate that perturbation of eCB signaling is an elegant potential causal effector in the regulation of maternal care and affiliative behaviors. These results are pertinent to better understanding how cannabinoid modulation contributes to the neurocircuitry of psychiatric disorders related to early-life adversity, such as depression, bipolar disorder and schizophrenia.^56, 96^

### Social communication

In humans, high concentrations of cannabinoid receptors are found in the left hemisphere cortical regions that are associated with verbal language function,^16, 18^ which suggests a role for eCB signaling in social communication. However, current findings only supports a role for eCB signaling in nonverbal motor-related aspects of social communication (that is, eye-gaze or body language). Genetic variants (that is, single-nucleotide polymorphisms) in CB1Rs are correlated with greater gaze duration to happy faces and are considered a putative endophenotype for communication deficits, particularly in ASD research.^99^ Animal models of social communication impairments demonstrate a direct connection to cannabinergic function, which interacts with the forkhead box (FOXP) protein family.^58^ Interestingly, FOXP are instrumental in ultrasonic vocalizations (USVs) production and vocal learning.^58, 100^ In rodents, USVs serve a communicatory function to elicit social interaction^61, 62^ or to share socially relevant information with known conspecifics.^63^ Moreover, USVs are developmental- and context-dependent^62^ relating identity, emotionality,^61, 62, 63^ receptivity to affiliative and sexual behaviors, and changes in the environment.^56^ Persistent activation of CB1R, via agonists or inhibitors of enzymatic degradation, decrease emission of USVs in rodent pups. Cannabinoids modulate USVs induced by maternal separation in pups,^61^ whereas in adult rodents, cannabinoids increase the emission of USVs when exposed to anxiogenic stimuli.^61, 63^ Moreover, CB1R knockout mice have low USV emission throughout development,^56^ which parallels the communication impairments associated with disorders such as ASD.^62^ For clinical populations with impairments in language acquisition, especially nonverbal patients with social deficits, cannabinergic modulation of USVs offers a novel and viable area of biomedical intervention in support of communication acquisition and development.^59, 60, 100^

## Conclusions

The diversity of social functioning deficits present in neuropsychiatric and neurodevelopmental disorders confounds the efficacy and specificity of treatment for affected individuals. However, the recent shift in psychiatric research towards examining the underlying dysregulated neural circuits of brain disorders has allowed consideration herein of novel aspects of the functionally diverse eCB signaling system. This article reviewed the eCB signaling system and the role of its components (that is, cannabinoid receptors, functional ligands) in varied, but convergent, RDoC domains of social functioning. From a molecular level of analysis, the cannabinoid system is implicated in negative and positive valence systems to restore homeostatic balance, increase the salience of reward learning and motivate social interactions. Behavioral and physiological effects of the eCB system are observed in cognitive systems. Social process systems show evidence of cannabinergic modulation, and as an emergent domain, rely on appropriate eCB function in the aforementioned RDoC domains. Although further work is needed to clarify the discrete roles of cannabinoid signaling in each RDoC domain, the extant evidence strongly supports the contribution of dysregulated cannabinoid signaling to the pathophysiology of social functioning impairments. With regard to the development of pharmacological interventions, although there is a diverse set of methods to enhance endogenous signaling or receptor stimulation *in vivo*, the lack of evidence on direct endocannabinoid (AEA or 2-AG)^66^ highlights the need for further research. Moreover, in the pursuit of precision health,^2^ although each component of the eCB system can be targeted for therapeutic development, appropriate targeting with respect to the intersection of the eCB system and a disorder's neurocircuitry must be carefully considered to achieve optimal outcomes. It is clear, though, that the eCB system holds exceptional promise as a biological marker of, and treatment target^6^ for, neuropsychiatric and neurodevelopmental disorders characterized by pronounced abnormalities in social functioning.

## Figures and Tables

**Table 1:** Endocannabinoid system component in RDoC domains

*RDoC construct*	*Mediating circuit*	*CB1R in circuitry*	*CB2R in circuitry*	*2-AG*	*AEA*
Anxiety	Interpret: BNST, HPC, amygdala Evaluate and response initiation: PFC, NAc, VTA, hypothalamus^28,29^	BNST^a^, ^a^HPC, amygdala, ^a^PFC, ^a^NAc medium spiny neurons, hypothalamus^a^,^5, 8, 18^	Microglia,^30^ VTA and NAc dopaminergic neurons^16, 31^	↑in brain → anxiolytic^30^	↓in brain → anxiogenic^30^
Chronic stress	HPA, amygdala–medial PFC–striatum^32, 33, 34^	^a^PFC, ^a^HPC, ^a^NAc, amygdala, HPA^a^,^19, 35^	Rats: amygdala, HPC, PFC, hypothalamus^25^	Amygdalar impairment in synthesis,^30^ ↑in PFC^36^	↓in amygdala (via↑FAAH)^30^
Motivation	Orbitofrontal cortex, amygdala, VTA, NAc^37, 38, 39^	Amygdala, ^a^HPC, VTA and ^a^NAc dopaminergic neurons^38, 40, 41^	VTA dopaminergic neurons^31, 40^	↑in NAc → ↑dopamine^38, 41, 42^	↑in NAc → ↑dopamine
Reward learning	VTA, NAc, PFC, hypothalamus, amygdala^40, 42^	^a^NAc medium spiny neurons, ^a^HPC, amygdala, VTA and ^a^NAc dopaminergic neurons, hypothalamus^a^, ^a^PFC^40, 42, 43^	VTA dopaminergic neurons^31^	↑in NAc → ↑dopamine^38, 41, 42^	↑in NAc → ↑dopamine
Declarative memory	PFC, amygdala, HPC (dentate gyrus, CA1, CA3), VTA, NAc^44^	^a^PFC, amygdala, GABAergic interneurons, ^a^HPC (dentate gyrus, CA1 and CA3).^18, 19^	HPC interneuron cholecystokinin (+) basket cells^45^	↓HPC CB1R binding → ↑GABA ^45, 46^	HPC^18^↑(via ↓FAAH) →↑acquisition^47, 48^
Working memory	PFC–parietal–cingulate–thalamus–NAc, HPC^49, 50^	^a^NAc medium spiny neuron, GABAergic hippocampal interneurons^46^	HPC^23^	↑HPC CB2R binding→ ↓encoding/consolidation^51^	↑ (via ↓FAAH) →↑acquisition^47, 48^
Affiliation and attachment	Amygdala, VTA, NAc, PVN, SON, HPA, BNST^52,53, 54^	PVN,^34^ SON,^55^ HPA,^a,13^ BNST^a^, amygdala, NAc^12, 56^	HPC^23, 56^	↑in HPC →↓ care^54, 57^ ↑in amygdala →↓play^36^	Play: ↑amygdala and NAc^12, 36^
Social communication	Amygdala, NAc, orbitofrontal cortex, cortico–basal ganglia–thalamo network^58^	HPC, amygdala, NAc ^59, 60^	Not yet known	Not yet known	↑ (via ↓FAAH) →↑USVs^61, 62, 63^

Abbreviations: AEA, anandamide; 2-AG, 2-arachidonoylglycerol; BNST, bed nucleus of the stria terminalis; FAAH, fatty acid amide hydrolase; GABA, glutamate and γ-aminobutyric acid; HPA, hypothalamus–pituitary–adrenal gland axis; HPC, hippocampus; NAc, nucleus accumbens; PFC, prefrontal cortex; PVN, paraventricular nucleus; RDoC, Research Domain Criteria; SON, supraoptic nucleus; USV, ultrasonic vocalization; VTA, ventral tegmental area.

CB1R is the most abundant G-protein-coupled receptor (^a^highest levels/densities, ^b^lowest) in the mammalian brain, contributing to cannabinergic effects on ‘movement, affective responding, cognition, temperature, appetite and neuroendocrine function'.^8, 18^

Human mRNA expression for a CB2R isoform has been found in amygdala, striatum, HPC, cortex and cerebellum,^25^ with rodents showing additional expression in the cerebral cortex, brain stem, thalamic nuclei and periaqueductal grey.^64^ AEA binding elicits sub-maximal receptor signaling, whereas 2-AG binds with much greater efficacy,^42^ necessary to produce robust phasic signaling responses.
